# Transcriptional Regulation of Drug Metabolizing CYP Enzymes by Proinflammatory Wnt5A Signaling in Human Coronary Artery Endothelial Cells

**DOI:** 10.3389/fphar.2021.619588

**Published:** 2021-05-17

**Authors:** Tom Skaria, Esther Bachli, Gabriele Schoedon

**Affiliations:** ^1^ Inflammation Research Unit, Division of Internal Medicine, University Hospital Zürich, Zürich, Switzerland; ^2^ School of Biotechnology, National Institute of Technology Calicut, Kerala, India; ^3^ Department of Medicine, Uster Hospital, Uster, Switzerland

**Keywords:** inflammation, Wnt5A, transcriptome profiling, pharmacokinetic pathways, cardiac vascular endothelial cells

## Abstract

Downregulation of drug metabolizing enzymes and transporters by proinflammatory mediators in hepatocytes, enterocytes and renal tubular epithelium is an established mechanism affecting pharmacokinetics. Emerging evidences indicate that vascular endothelial cell expression of drug metabolizing enzymes and transporters may regulate pharmacokinetic pathways in heart to modulate local drug bioavailability and toxicity. However, whether inflammation regulates pharmacokinetic pathways in human cardiac vascular endothelial cells remains largely unknown. The lipid modified protein Wnt5A is emerging as a critical mediator of proinflammatory responses and disease severity in sepsis, hypertension and COVID-19. In the present study, we employed transcriptome profiling and gene ontology analyses to investigate the regulation of expression of drug metabolizing enzymes and transporters by Wnt5A in human coronary artery endothelial cells. Our study shows for the first time that Wnt5A induces the gene expression of CYP1A1 and CYP1B1 enzymes involved in phase I metabolism of a broad spectrum of drugs including chloroquine (the controversial drug for COVID-19) that is known to cause toxicity in myocardium. Further, the upregulation of CYP1A1 and CYP1B1 expression is preserved even during inflammatory crosstalk between Wnt5A and the prototypic proinflammatory IL-1β in human coronary artery endothelial cells. These findings stimulate further studies to test the critical roles of vascular endothelial cell CYP1A1 and CYP1B1, and the potential of vascular-targeted therapy with CYP1A1/CYP1B1 inhibitors in modulating myocardial pharmacokinetics in Wnt5A-associated inflammatory and cardiovascular diseases.

## Introduction

Inflammation is the first line innate immune response to protect the host from infections or tissue injury. It involves highly coordinated interaction of antigen-activated immune cells and their soluble inflammatory products with vascular endothelial cells, inducing a procoagulant, immune cell adhesive and hyperpermeable phenotype in vascular endothelial cells, followed by the movement of immune cells, soluble inflammatory mediators and other plasma proteins across vascular endothelial cells to the site of infection or injury to minimize tissue damage (Pober and Sessa, 2007). Although an orchestrated inflammatory response is crucial for efficient immunity, uncontrolled or sustained inflammation becomes pathogenic and causes tissue destruction, impairs organ function and affects drug pharmacokinetics (Morgan, 2009; Netea et al., 2017). The importance of deregulated immune defense is obvious even in the current pandemic COVID-19 where, endotheliitis, for example in the heart leads to local thrombosis (Varga et al., 2020). Drug pharmacokinetics is affected when locally produced proinflammatory cytokines enter systemic circulation and exert inflammatory responses in hepatocytes, enterocytes and renal tubular epithelium, which represent the classical sites for action of drug metabolizing enzymes and transporters. It was shown that proinflammatory cytokines such as interleukin (IL)-1β and tumor necrosis factor-α downregulate the transcription of cytochrome P450 (CYP) enzymes involved in phase I oxidative metabolism, and membrane protein drug transporters such as Organic Anion Transporting Polypeptide (OATP)- 1 and 2 in hepatocytes, enterocytes and renal tubular epithelium. This results in decreased hepatic clearance and enhanced oral bioavailability increasing the incidence of adverse events. In case of prodrugs activated by metabolism, decreased activities of CYP enzymes may reduce their therapeutic efficiency (Morgan, 2009; König et al., 2013; Wu and Lin, 2019).

Emerging evidences indicate that cardiac vascular endothelial cell expression of drug and xenobiotic metabolizing enzymes and transporters, involved in local metabolic homeostasis, can also modulate pharmacokinetics in the heart muscle. It is shown that organic cation transporter novel type 2, a sodium dependent transport protein for carnitine, is expressed and localized in normal cardiac endothelial cells. Its cardiac expression regulates cardiac delivery of spironolactone or mildronate during congestive heart failure. High variability in its cardiac expression among individuals is linked to variable response to its substrate drugs in clinical setting (Grube et al., 2006). Similarly, multidrug resistance protein 1 (MDR1), a drug efflux pump, is expressed in normal cardiac endothelial cells, and modulates myocardial uptake of its substrates talinolol and celiprolol. Further, cardiac vascular endothelial expression of MDR1 may mediate inter-individual variability observed for the positive inotropic effects of its another substrate digoxin (Meissner et al., 2002; Hausner et al., 2019). In addition to their effects on drugs, cardiac vascular endothelial expression of drug/xenobiotic metabolizing enzymes and transporters also modulates disease modifying endobiotic transformations. Blocking CYP2C9 activity using sulfaphenazole decreased experimentally induced infarct size and post-ischemic vascular superoxide generation, and enhanced post-ischemic coronary flow (Granville et al., 2004; Hunter et al., 2005; Michaud et al., 2010). All these recent findings clearly reveal a critical role for the intrinsic activity of cardiac vascular endothelial cell-expressed drug metabolizing enzymes and transporters in modulating drug and xenobiotic concentrations in myocardium. However, there has been no study performed yet to investigate whether inflammation that has an established role in affecting pharmacokinetics pathways in hepatocytes, enterocytes and renal tubular epithelium (Morgan, 2009; König et al., 2013; Wu and Lin, 2019), regulates the expression of drug and xenobiotic metabolizing enzymes and transporters in human cardiac vascular endothelial cells. In precision medicine, a comprehensive knowledge of the regulation of pharmacokinetic pathways in vascular endothelial cells by specific inflammatory mediators is crucial for developing vascular-targeted therapy to reduce inter-individual variability in drug response and local and systemic toxicity (Eelen et al., 2015; Fatunde and Brown, 2020; Glassman et al., 2020).

In the present study, we employed whole genome expression profiling to investigate whether Wnt5A, an emerging inflammatory mediator in vascular system (Blumenthal et al., 2006; Pereira et al., 2008; Schulte et al., 2012; Skaria and Schoedon, 2017; Choi et al., 2020), regulates the expression of drug metabolizing enzymes and transporters in immunocompetent, primary, human coronary artery endothelial cells (HCAEC; Skaria et al., 2017; Skaria et al., 2019). In our present study, Wnt5A treatments of HCAEC were conducted for 4 h. We chose 4 h treatment in this study because several previous independent studies established that the effects of Wnt5A are time-dependent in different cell types (Valencia et al., 2014; Shojima et al., 2015; Huang et al., 2017). Here, we find that in HCAEC, Wnt5A critically modulates myocardium-specific pharmacokinetic pathways by upregulating the transcription of CYP enzymes that are known to metabolize a broad spectrum of drugs including those used in immune system and cardiovascular diseases.

## Materials and Methods

### Primary Cell Culture

HCAEC were propagated, and treated with vehicle (sterile, pyrogen free, 0.1% human serum albumin in 0.9% NaCl) and recombinant human/mouse Wnt5A (250 ng/ml, R&D systems) alone or combined with recombinant human IL-1β (20 U/ml, PeproTech) for 4 h as described (Skaria et al., 2017; Skaria et al., 2019) (detailed in Supplementary Material). Specific information about vascular endothelial cell characterization is provided in Supplementary Material.

### Whole Genome Expression Profiling and Gene Ontology Analysis

Differential gene expression profiling using microarray analysis, and scanning, feature extraction, and data normalization of microarrays were performed using established methods (Skaria et al., 2017; Skaria et al., 2019) (detailed in Supplementary Material). Complete data sets of Wnt5A and Wnt5A/IL-1β combination transcriptomes in HCAEC are accessible in the NCBI GEO data repository through accession numbers GSE145987 and GSE62281, and GSE146691 respectively (refer Supplementary Material for particulars about accession numbers). Linear-lowess normalized microarray data were further analyzed using GeneSpring GX 9.0 Software (Agilent Tech. Inc.), and gene ontology analysis to identify drug and xenobiotic metabolism pathways significantly (*p* < 0.05) enriched in microarray data were performed using MetaCoreTM GeneGO software (Thomson Reuters, http://portal.genego.com) as described (Skaria et al., 2017; Skaria et al., 2019) with modifications (detailed in Supplementary Material).

## Results

**FIGURE 1:**
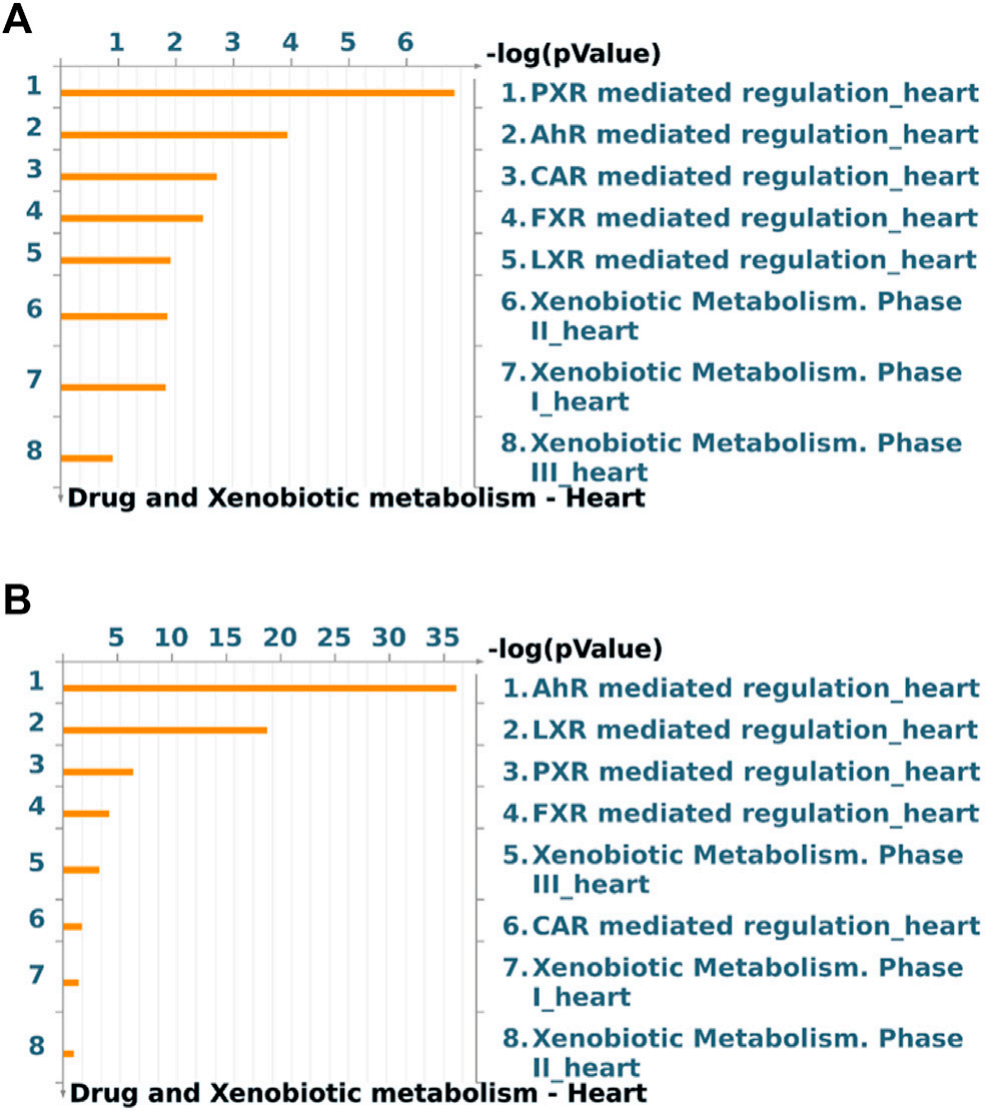
Drug and xenobiotic metabolism pathways most significantly (*p* < 0.05) regulated by 4 h Wnt5A **(A)** and Wnt5A/IL-1β combination **(B)** treatment in HCAEC. Pathways represented as histograms are ranked by the–log value (*p* value). Data are from 3 independent experiments.

Global gene expression profile of 4 h Wnt5A treated HCAEC was compared with that of vehicle-treated HCAEC by whole human genome microarrays. Genes of Wnt5A-treated HCAEC which are significantly differentially regulated after linear-lowess normalization (refer Supplementary Material) and consistently showing at least two-fold change in expression in subsequent GeneSpring analysis compared with vehicle-treated HCAEC were identified (Supplementary Table S1) and screened with MetaCoreTM GeneGO software for their involvement in regulating drug and xenobiotic metabolism pathways. PXR mediated regulation_heart, AhR mediated regulation_heart, CAR mediated regulation_heart, FXR mediated regulation_heart, LXR mediated regulation_heart, Xenobiotic Metabolism- phase II_heart, Xenobiotic Metabolism- phase I_heart, and Xenobiotic Metabolism- phase III_heart were the drug and xenobiotic metabolism pathways significantly enriched in 4 h Wnt5A transcriptome of HCAEC (Figure 1A). Genes of these statistically significant, enriched pathways upregulated by Wnt5A include those encoding intracellular enzymes CYP1A1 and CYP1B1 involved in phase I oxidation, and the transmembrane peptide SLCO2B1 transporting large hydrophobic organic anions, cations and neutral compounds (Table 1). Abundant protein expression of CYP1A1, CYPB1 and SLCO2B1 has been verified in human myocardium (Table 1). CYP1A1 is reported to metabolize compounds such as the antiarrhythmic drug amiodarone, antimicrobial erythromycin, antimalarial and immunomodulatory chloroquine (controversial in use against COVID-19 as an agent preventing the entry of SARS-CoV-2 through ACE2 receptor), nonsteroidal anti-inflammatory diclofenac, anti-psychotic haloperidol, steroid hormone estradiol and the chemotherapeutic agent daunorubicin in humans (Supplementary Table S2). Chloroquine, with active metabolites and long half-life, can prolong the QT interval that could trigger ventricular arrhythmias including torsades de pointes (Kamp et al., 2020). Haloperidol is still used in treating delirium in septic patients admitted in ICU and is associated with QT interval prolongation (Huffman and Stern, 2003). CYP1B1 metabolizes drugs including the anticancer procarbazine, theophylline and the most prescribed cholesterol lowering drug rosuvastatin in humans (Supplementary Table S2). SLCO2B1 can transport drugs such as the leukotriene receptor antagonist montelukast used for asthma, the antirheumatic, immunosuppressive sulfasalazine and a number of drugs acting on the cardiovascular system such as aliskiren, antihypertensive drugs of the sartan group (telmisartan) and a number of cholesterol lowering agents (rosuvastatin, atorvastatin, pravastatin) (Supplementary Table S2). Genes of significantly enriched pharmacokinetic pathways downregulated by Wnt5A include UDP-glucuronosyltransferases (UGT)-1A4 and 1A6 (Table 1) involved in phase II drug metabolism of drugs such as the anticonvulsant lamotrigine and anti-atherosclerotic/analgesic aspirin respectively (Bigler et al., 2001; Reimers et al., 2016).

**TABLE 1:** Genes of statistically significant (*p <* 0.05) drug and xenobiotic metabolism pathways regulated by 4 h Wnt5A treatment in HCAEC. Data are from 3 independent array experiments.

Gene symbol	Protein name	Class	Regulation
CCNC^a^ ^,^ ^b^ ^,^ ^c^ ^,^ ^e^ ^,^ ^i^	Cyclin C	Generic binding protein	Down
CEACAM3^b^ ^,^ ^i^	Carcinoembryonic antigen-related cell adhesion molecule 3	Generic protein	Down
CHI3L1^b^ ^,^ ^e^ ^,^ ^i^	Chitinase-3-like protein 1	Generic enzyme	Down
CYP1A1^a^ ^,^ ^b^ ^,^ ^c^ ^,^ ^g^ ^,^ ^i^	Cytochrome P450 1A1	Generic enzyme	Up
CYP1B1^b^ ^,^ ^e^ ^,^ ^g^ ^,^ ^i^	Cytochrome P450 1B1	Generic enzyme	Up
DCHS2^b^	Protocadherin-23	Generic binding protein	Down
EDNRA^a^ ^,^ ^b^ ^,^ ^e^ ^,^ ^i^	Endothelin-1 receptor	G protein-coupled receptor	Up
EDNRB^a^ ^,^ ^b^ ^,^ ^e^ ^,^ ^i^	Endothelin receptor type B	G protein-coupled receptor	Down
GNAO1^b^ ^,^ ^i^	Guanine nucleotide-binding protein G(o) subunit alpha	G-alpha	Down
HNF4A^a^ ^,^ ^d^	Hepatocyte nuclear factor 4-alpha	Transcription factor	Down
IGF2^a^ ^,^ ^i^	Insulin-like growth factor II	Receptor ligand	Down
IL2^b^	Interleukin-2	Receptor ligand	Down
ITGA4^b^ ^,^ ^i^	Integrin alpha-4	Generic receptor	Up
ITGB6^b^	Integrin beta-6	Generic receptor	Down
KCTD12^b^ ^,^ ^e^ ^,^ ^i^	BTB/POZ domain-containing protein KCTD12	Voltage-gated ion-channel	Up
KLK12^c^ ^,^ ^e^	Kallikrein-12	Generic protease	Down
LILRB4^e^ ^,^ ^i^	Leukocyte immunoglobulin-like receptor subfamily B member 4	Generic receptor	Down
MS4A2^e^ ^,^ ^i^	High affinity immunoglobulin epsilon receptor subunit beta	Generic receptor	Up
NDUFS7^b^ ^,^ ^e^ ^,^ ^i^	NADH dehydrogenase [ubiquinone] iron-sulfur protein 7, mitochondrial	Generic enzyme	Down
OR4C16^a^	Olfactory receptor 4C16	G protein-coupled receptor	Down
OR4D2^a^	Olfactory receptor 4D2	G protein-coupled receptor	Down
OR4F4^a^	Olfactory receptor 4F4	G protein-coupled receptor	Down
OR6Y1^a^	Olfactory receptor 6Y1	G protein-coupled receptor	Up
OR8J1^a^	Olfactory receptor 8J1	G protein-coupled receptor	Up
OR9G4^a^	Olfactory receptor 9G4	G protein-coupled receptor	Down
PSG5^b^	Pregnancy-specific beta-1-glycoprotein 5	Generic protein	Up
SLC16A2^h^ ^,^ ^i^	Monocarboxylate transporter 8	Transporter	Up
SLCO2B1^a^ ^,^ ^b^ ^,^ ^e^ ^,^ ^h^ ^,^ ^i^	Solute carrier organic anion transporter family member 2B1	Transporter	Up
SYT6^b^	Synaptotagmin-6	Generic receptor	Down
TCTN3^b^ ^,^ ^i^	Tectonic-3	Generic protein	Down
TFAP2D^d^	Transcription factor AP-2-delta	Transcription factor	Down
TIMP1^d^ ^,^ ^e^ ^,^ ^i^	Metalloproteinase inhibitor 1	Generic binding protein	Down
UGT1A4^a^ ^,^ ^b^ ^,^ ^c^ ^,^ ^d^ ^,^ ^f^	UDP-glucuronosyltransferase 1-4	Generic enzyme	Down
UGT1A6^a^ ^,^ ^b^ ^,^ ^c^ ^,^ ^d^ ^,^ ^f^	UDP-glucuronosyltransferase 1-6	Generic enzyme	Down

^a^Genes regulated in PXR mediated regulation_heart.

^b^ Genes regulated in AhR mediated regulation_heart.

^c^ Genes regulated in CAR mediated regulation_heart.

^d^ Genes regulated in FXR mediated regulation_heart.

^e^ Genes regulated in LXR mediated regulation_heart.

^f^ Genes regulated in Xenobiotic Metabolism. Phase II_heart.

^g^ Genes regulated in Xenobiotic Metabolism. Phase I_heart.

^h^ Genes regulated in Xenobiotic Metabolism. Phase III_heart.

^i^ Protein expression verified in normal human myocardium as shown in The Human Protein Atlas (accessed on 03.08.2020).

During inflammatory diseases such as sepsis and atherosclerosis, vascular endothelial cells may not be exposed to a single inflammatory mediator, rather, different inflammatory mediators such as Wnt5A and the prototypic proinflammatory prothrombotic proatherogenic IL-1β simultaneously act paracrinically on vascular endothelial cells and their crosstalk may modulate inflammatory responses in vascular endothelial cells (Pereira et al., 2008; Bhatt et al., 2012; Schulte et al., 2012; Gatica-Andrades et al., 2017; Skaria and Schoedon, 2017). This prompted us to test whether the regulation of expression of CYP enzymes, known to metabolize broad spectrum of drug substrates (Supplementary Table S2) and found regulated by sole Wnt5A treatment in this study (Table 1; Supplementary Table S1), is preserved during crosstalk between Wnt5A and IL-1β in HCAEC. CYP1A1 and CYP1B1 remained upregulated by Wnt5A/IL-1β combination treatment in HCAEC (Supplementary Tables S3, S4). Further, Wnt5A/IL-1β signaling interaction upregulated the gene encoding an additional member of CYP enzyme family CYP7A1 (Supplementary Tables S3, S4). Protein expression of CYP7A1 has been verified in normal human myocardium (Supplementary Table S4), however its substrates in humans remain largely unidentified. Moreover, Wnt5A/IL-1β combination treatment significantly enhanced enrichment of genes in AhR mediated regulation_heart and LXR mediated regulation_heart pharmacokinetic pathways in HCAEC compared with Wnt5A or IL-1β alone treatments (Figure 1B; Supplementary Figure S1; Supplementary Table S4).

## Discussion

Transcriptional downregulation of expression of drug metabolizing enzymes and transporters by the systemic action of proinflammatory mediators in hepatocytes, enterocytes and renal tubular epithelium is an established mechanism affecting pharmacokinetics during inflammation (Morgan, 2009; Wu and Lin, 2019). Additionally, increasing evidences indicate that vascular endothelial expression of drug metabolizing enzymes and transporters may regulate pharmacokinetic pathways in heart to modulate local drug bioavailability and toxicity in humans (Meissner et al., 2002; Grube et al., 2006; Hausner et al., 2019). However, whether inflammatory activation regulates pharmacokinetic pathways in human cardiac vascular endothelial cells remained largely unknown. This study investigated for the first time the regulation of expression of drug metabolizing enzymes and transporters by proinflammatory mediator Wnt5A in human coronary artery endothelial cells. It reveals that Wnt5A upregulates the mRNA expression of CYP1A1 and CYP1B1; enzymes with known role in phase I metabolism of a broad of spectrum of drugs and their protein expression established in human myocardium. Further, it reveals that upregulated CYP1A1 and CYP1B1 expression is preserved during inflammatory crosstalk between Wnt5A and proinflammatory IL-1β in human coronary artery endothelial cells. This novel finding from human vascular endothelial cells isolated from coronary artery, a primary cell system retaining original tissue characteristics (Franscini et al., 2004; Skaria et al., 2017; Skaria et al., 2019), is in accordance with previous findings that proinflammatory cytokines, in contrast to their suppressive effects on drug metabolizing pathways in hepatocytes (Morgan, 2009; Wu and Lin, 2019), stimulate the transcription of CYP enzymes in extrahepatic cell systems (Smerdová et al., 2014; Alhouayek et al., 2018).

Previous studies showed that CYP1A1, involved in transformation of xenobiotics to toxic metabolites, also metabolizes a broad spectrum of drugs and consequently account for drugs’ adverse effects. CYP1A1 metabolizes the class III antiarrhythmic drug amiodarone to desethyl amiodarone, the latter causes toxicity in multiple organs (Wu et al., 2016). Another substrate of CYP1A1 is the macrolide erythromycin used as an antiinfection agent or for gastrointestinal disease in ICUs (Zhou et al., 2019). Overexpression of CYP1A1 by Wnt5A may enhance erythromycin’s metabolism and thus affects its half-life leading to the persistence of infection and lack of drug efficiency. Likewise, enhanced CYP1A1 activity may increase the clearance of theophylline used in treatment of obstructive pulmonary disease (Sarkar and Jackson, 1994). In pathological states such as sepsis, cardiac arrhythmia associated with hypertension and chronic obstructive lung diseases, Wnt5A signaling is activated in the cardiovascular system (Pereira et al., 2008; Schulte et al., 2012; Daud et al., 2016; Abraityte et al., 2017a; Baarsma et al., 2017; Abraityte et al., 2017b). This stimulates further investigations to determine whether circulating Wnt5A concentration correlates with myocardial CYP1A1/CYP1B1 activity, drug availability and cardiotoxicity in these diseases.

A previous study showed transcriptional downregulation of CYP1B1 and upregulation of CYP1A1 in endothelial cells with homozygous null mutation of the β-catenin gene, isolated from E9.5 embryos (Ziegler et al., 2016). Several independent, previous studies established that endothelial cells derived from embryo exhibit high plasticity and therefore significantly differs in morphology and in response to signaling molecules compared with adult human vascular endothelial cells (Risau, 1995; Invernici et al., 2005; Földes et al., 2010). Accordingly, the aforesaid study involving endothelial cells from E9.5 embryos additionally demonstrated upregulation of CYP1B1 and unaltered expression of CYP1A1 in response to canonical Wnt3A conditioned medium by mouse brain microvascular endothelial cells. Furthermore, the aforesaid study showed that mouse brain microvascular endothelial cells respond to non-canonical Wnt5A-conditioned medium by decreasing CYP1B1 transcription (Ziegler et al., 2016). As the above study itself and several other previous studies proved, Wnt ligands regulate multiple signaling pathways depending on the availability of specific receptors and other mediators of the signaling pathway, cellular conditions, and the presence of natural inhibitors like sFRP and WIF1 (Pukrop and Binder, 2008; Kikuchi et al., 2012; Ziegler et al., 2016). In this manuscript, we show the transcriptional upregulation of CYP1A1 and CYP1B1 by exogenous Wnt5A in primary, coronary artery endothelial cells derived from adult human myocardium. Primary, coronary artery endothelial cells derived from adult human myocardium was chosen in this study to assess how Wnt5A regulates the drug metabolizing potential of myocardial vasculature because it is well established that vascular endothelial cells from different anatomical locations like brain and heart significantly differ in their response to signaling molecules (Aird, 2007).

In precision medicine, therapeutically modulating a specific characteristic of inflamed vascular endothelial cells by vascular-targeted nanocarriers is a potential strategy to reduce inter-individual variability in drug response and local toxicity (Eelen et al., 2015; Glassman et al., 2020). Therefore, targeting vascular endothelial CYP1A1 and CYP1B1 by their inhibitors loaded in nanocarriers conjugated with affinity ligands of inflamed endothelial markers may be a potential strategy to modulate myocardial pharmacokinetics of CYP1A1 and CYPB1 substrates in diseases associated with Wnt5A. Further, a precise knowledge on the regulation of pharmacokinetic pathways by specific inflammatory mediators may enable adapting drug dosage regimens according to the changes in inflammatory status of patients (Morgan, 2009) as has been postulated even in the case of emerging COVID-19 pandemic (El-Ghiaty et al., 2020). Most interesting in the latter context is the fact that medication proposed to target ACE2 (Wang et al., 2020) are metabolized through CYP1A1, and our observation that CYP1A1 but not ACE2 expression, is a target for transcriptional modulation by Wnt5A. Most recently, Wnt5A has been found significantly elevated in severe cases of COVID-19 (Choi et al., 2020), and endotheliitis was observed as major pathology in severe COVID-19 (Varga et al., 2020). Therefore, while targeting ACE2 with drugs that are substrates for CYP1A1, the modulation of those drugs’ pharmacokinetics by Wnt5A-inflamed cardiac vascular endothelial cells might occur and must be a focus of future studies. Drug metabolizing enzymes and transporters are also transcriptionally regulated by the xenobiotic receptors (XR) such as constitutive androstane receptor (CAR), the pregnane X receptor (PXR) and the aryl hydrocarbon receptor (AhR), which are mainly expressed in the liver (Mackowiak and Wang, 2016). It is noteworthy that their expression has been reported in vascular endothelial cells (Agbor et al., 2011). Therefore, whether their activation by endogenous compounds and drugs or xenobiotics transcriptionally regulate the phase I and phase II metabolizing enzymes or drug transporters in vascular endothelial cells during inflammation warrants further investigations. Moreover, in light of emerging evidences indicating that cytokine signaling pathways activate XR even in the absence of their xenobiotic activators (Mackowiak and Wang, 2016), the ability of Wnt5A/IL-1 signaling pathways to mediate XR activation in the absence of drugs/metabolites in pathological states needs to be further investigated.

In conclusion, this study shows for the first time that the proinflammatory mediator Wnt5A upregulates human coronary artery endothelial expression of CYP1A1 and CYP1B1 enzymes involved in phase I metabolism of a broad spectrum of drugs. Upregulated CYP1A1 and CYP1B1 expression is preserved during inflammatory crosstalk between Wnt5A and proinflammatory IL-1β in human coronary artery endothelial cells. These preliminary findings presented in this brief research report stimulate further studies on the critical roles of drug metabolizing potential of Wnt5A-inflamed adult human myocardial vasculature and the therapeutic benefits of vascular-targeted inhibitors of CYP1A1/CYP1B1 in modulating myocardial pharmacokinetics in Wnt5A-associated inflammatory diseases.

## Data Availability

The datasets presented in this study can be found in online repositories. The names of the repository/repositories and accession number(s) can be found in the article/Supplementary Material.
