# Supplementary material for: Transcriptional Regulation of Drug Metabolizing CYP Enzymes by Proinflammatory Wnt5A Signaling in Human Coronary Artery Endothelial Cells
Source: Front Pharmacol. 2021 May 17;12:619588. doi: 10.3389/fphar.2021.619588 (PMC8165381; doi:10.3389/fphar.2021.619588)
Supplement: Supplementary file 1 [file datasheet1.pdf]

Supplementary Material

Transcriptional regulation of drug metabolizing CYP enzymes by proinflammatory Wnt5A signaling in human coronary artery endothelial cells

Tom Skaria[1, 2], Esther Bachli[3], Gabriele Schoedon[1*]

[1]Inflammation Research Unit, Division of Internal Medicine, University Hospital Zürich, Zürich, Switzerland, [2]School of Biotechnology, National Institute of Technology Calicut, Kerala, India, [3]Department of Medicine, Uster Hospital, Uster, Switzerland.

*Correspondence

Gabriele Schoedon

Email: klinsog@usz.uzh.ch

Supplementary Methods

Supplementary Results

Supplementary Table 1. Genes regulated (≥2 fold up or down) by 4 h Wnt5A treatment in HCAEC. Data are from 3 independent array experiments.

Supplementary Table 2. Substrate compounds of CYP1A1 and CYP1B1 retrieved from cytochrome P450 database SuperCYP (http://bioinformatics.charite.de/supercyp/index.php?site=home, accessed through 'CYP-drug interaction' on 03.08.2020), and SLCO2B1 substrates retrieved from metabolism and transport database Metrabase (http://www-metrabase.ch.cam.ac.uk/, accessed through 'search by protein' on 03.08.2020).

Supplementary Table 3. Genes regulated (≥2 fold up or down) by 4 h Wnt5A/IL-1β combination treatment in HCAEC. Data are from 3 independent array experiments.

Supplementary Table 4. Regulated genes of drug and xenobiotic metabolism pathways significantly *($P<0.05$)* enriched in 4 h Wnt5A+IL-1β transcriptome of HCAEC. Data are from 3 independent array experiments.

Supplementary Figure 1. Drug and xenobiotic metabolism pathways most significantly *($P<0.05$)* regulated by 4 h IL-1β treatment in HCAEC. Pathways represented as histograms are ranked by the –log value (P value). This figure is generated by reanalysing data published in *Skaria, T., Bachli, E., and Schoedon, G. (2019). Gene Ontology Analysis for Drug Targets of the Whole Genome Transcriptome of Human Vascular Endothelial Cells in Response to Proinflammatory IL-1. Frontiers in Pharmacology 10*. The purpose of reanalysing data from above-said study was to identify the myocardial drug and xenobiotic metabolism pathways regulated by 4 h IL-1β treatment (which was not contained in previous study) that served as an additional positive control for comparing the effects of Wnt5A/ IL-1β combination treatment with sole Wnt5A treatment in HCAEC.

Supplementary Methods

Primary cell culture

HCAEC (Cat. No. CC-2585, Clonetics, Lonza) were cultured in EGM-2MV Single Quots and 5% FBS (Clonetics, Lonza)-enriched EBM-2 medium (Clonetics, Lonza) in a Class 100 HEPA air filtered SteriCult system (Fisher Scientific, Switzerland) as described (Skaria et al., 2017; Skaria et al., 2019). HCAEC used in the present study were tested positively and functionally for CD31 (PECAM-1), CD105 (endoglin), von Willebrand Factor VIII, and acetylated low-density lipoprotein uptake as confirmed by the manufacturer (Lonza, Cell Systems). Masked low-level contamination in cultures were prevented by using antibiotic-free culture medium during the entire study period. Experiments were carried out using HCAEC from passages 3-6 only. HCAEC monolayers were treated with vehicle (sterile pyrogen free 0.1% human serum albumin in 0.9% NaCl) and recombinant human/mouse Wnt5A (250 ng/mL, Cat. No. 645-WN, R&D systems) alone or combined with recombinant human IL-1β (20 U/mL, Cat. No. 200-01B, PeproTech) for 4 h. All pipetting steps were carried out using Biopure ep Dualfilter T.I.P.S. sterile filter tips (Eppendorf) or TipOne aerosol barrier sterile filter pipet tips (USA Scientific).

Whole genome expression profiling and gene ontology analysis

Differential gene expression analysis by competitive two-colour hybridization of cRNA probes onto 4×44K Human Whole Genome Oligonucleotide microarrays (Agilent Technologies) was conducted as described (Skaria et al., 2017; Skaria et al., 2019). Following isolation of total RNA using RNeasy Mini Kit (Qiagen, Basel, Switzerland), RNA was quantified by Nanodrop spectrophotometry. RNA integrity was measured with the Agilent 2100 Bioanalyzer System (Agilent Tech., Basel, Switzerland). Only RNA samples with a RNA integrity number (RIN) >9 were used for the microarray experiments. From each of treated samples, 500 ng of total RNA were subjected to reverse transcription and labelling with Cy3- and Cy5-CTP employing the two-colour Quick Amp Labelling Kit (Agilent Tech., Switzerland) comprising internal control probes and spike in's (labelled Spike A mix with Cy-3 and Spike B mix with Cy-5) to control reaction performance and background normalization of arrays. cRNA fragmentation and hybridization onto the Human GE 4×44K V2 Microarray chips were conducted in accordance with the Quick Amp Labelling protocol (Agilent Techno., version 5.7, 2008). Ozone-induced deterioration of cyanine dyes were avoided by washing then array chips with stabilization and drying solution (Agilent Tech., Switzerland). Scanning, feature extraction and pre-processing in conjunction with data normalization of the microarrays were conducted using the Microarray Scanner and Feature Extraction Software 10.7 (Agilent Tech. Inc.) employing default settings for Agilent 4×44 K two-colour arrays. Spot value normalization was performed with the default linear-lowess normalization. Log ratios and P value Log ratios, calculated based on extensive error model and pixel level statistics computed from the feature and background for each spot, were used to indicate significant differential regulation of genes of the whole transcriptome after linear-lowess normalization and provided in all microarray datasets deposited in NCBI GEO database (available with accession numbers GSE145987, GSE62281 and GSE146691).

The accession GSE145987, presented for the first time in this manuscript, contains 2 independent microarray experiments of HCAEC treated with Wnt5A for 4 h (2 independent replicates). The accession GSE146691, presented for the first time in this manuscript, contains 3 independent microarray experiments of HCAEC treated with Wnt5A/IL-1 combination for 4 h. The accession GSE62281 contains the third independent microarray data of HCAEC treated with Wnt5A for 4 h (third independent replicate). Of these three accession numbers, only the accession GSE62281 is associated with a previous publication (Skaria et al., 2017). However,

the aforesaid previous research article (Skaria et al., 2017) contained analysis/description of the microarray data set of only 8 h Wnt5A alone and 8 h IL-1 alone treatment of HCAEC along with several other experiments addressing the effects of chronic Wnt5A treatment on endothelial barrier function pathway. It has to be noted that though microarray data of single 4 h Wnt5A treatment experiment has been contained in the GSE62281, the aforesaid previous publication (Skaria et al., 2017) contained neither analysis nor description of the 4 h Wnt5A alone or combined Wnt5A/IL-1 microarray data. Furthermore, the aforesaid previous study (Skaria et al., 2017) did not involve any gene ontology analysis for the direct drug/ drug metabolising targets even for the 8 h Wnt5A- or 8 h combined Wnt5A/IL-1-treated whole genome transcriptome of HCAEC.

To identify genes that were consistently regulated ≥ 2-fold in their expression in three independent microarray experiments in each treatment group, the pre-processed microarray data (that underwent linear-lowess normalization and error estimates) were further analysed using GeneSpring GX 9.0 Software (Agilent Tech. Inc.) with default settings for two-colour arrays. Genes regulated ≥2-fold in their expression as found from the second line GeneSpring analysis (Supplementary Tables S1 and S3) were subjected to gene ontology analysis of drug and xenobiotic metabolising enzymes using Metacore GeneGO software version 6.32.69020 (Thomson Reuters, http://portal.genego.com) after setting a P value < 0.05 and FDR cut off (0.05).

References

Skaria, T., Bachli, E., and Schoedon, G. (2017). Wnt5A/Ryk signaling critically affects barrier function in human vascular endothelial cells. *Cell Adh Migr* 11**,** 24-38.

Skaria, T., Bachli, E., and Schoedon, G. (2019). Gene Ontology Analysis for Drug Targets of the Whole Genome Transcriptome of Human Vascular Endothelial Cells in Response to Proinflammatory IL-1. *Frontiers in Pharmacology* 10.

Supplementary Results

Supplementary Table 1. Genes regulated (≥2 fold up or down) by 4 h Wnt5A treatment in HCAEC. Data are from 3 independent array experiments.

Upregulated genes	Downregulated genes
ALDH1L1	A2ML1
ATAD3C	AATK
C1orf172	ABCB5
C2CD4A	ACOT6
C6	ACSM5
CBLN4	AIM2
CEACAM21	ALS2CR12
CELA3A	ANKRD30B
CLCA2	APOL6
CNTN2	ATXN3L
CYP1A1	BCL11A
CYP1B1	BCOR
DDO	BEST1
DNM1L	BHMT
DSCR6	BIN2
EDNRA	C10orf71
EFCAB9	C10orf81
ELK1	C12orf40
ETV3	C12orf54
FAM90A7	C12orf61
FLJ10038	C14orf23
FLJ39080	C14orf86
GSG1	C18orf34
GYPE	C1orf130
HEATR1	C1orf141
HOMER1	C1orf192
HYALP1	C1orf227
IL22RA2	C20orf71
ITGA4	C2orf72
KCNS2	C5orf60
KCTD12	C6orf112
KIR3DL3	C7orf66
KRT24	C7orf72
LOC100130428	C9orf66
LOC100131702	CAGE1
LOC100507699	CCL28
LOC283588	CCNC
LOC440386	CD200R1
MAGEC2	CDH23
MIXL1	CEACAM3
MS4A2	CHI3L1
MYL10	CHRM3
MYO3A	CHRNA4
MYOD1	CLDN19
NEUROD2	CLEC4M
OR6Y1	CLRN2
OR8J1	CMAHP
PADI6	CNKSR2
PGC	CSMD2
POU5F2	CTAGE5
PSG5	DAPL1
PTAR1	DCHS2
PTCD3	DCLK1
PTPRC	DEFB113
RNASE6	DKFZp547J222
ROBO2	DMGDH
S100A14	DNAH17
SCARNA23	DTHD1
SGCD	EDNRB

SHANK2	EIF3E
SHISA2	ENO4
SLA	EPAG
SLC10A7	EPO
SLC16A2	F13A1
SLC24A4	FABP12
SLC39A12	FAM163A
SLC4A5	FBP2
SLCO2B1	FGF16
TBX21	FHAD1
TDRD6	FLJ46120
TLE1	FMN2
TMEM31	FRY
TTC30A	FSTL4
UMODL1	GAFA1
VIT	GATA4
ZNF780A	GCNT3
ZRANB3	GCNT7
	GFAP
	GFRAL
	GNAO1
	GPR6
	GPR77
	GRIP2
	GUCY2C
	HIST1H2BA
	HMCN2
	HNF4A
	HOXD9
	HPGD
	HPSE2
	HSPB7
	IGF2
	IKZF1
	IL16
	IL2
	IL31
	ISL2
	ITGAD
	ITGB6
	KAZN
	KCNV1
	KIAA1875
	KLHL15
	KLK12
	KRTAP19-4
	KRTAP7-1
	LAIR2
	LILRB4
	LIPC
	LOC100128077
	LOC100128164
	LOC100128356
	LOC100128402
	LOC100129072
	LOC100129119
	LOC100129413
	LOC100130157
	LOC100130255
	LOC100130452
	LOC100130701
	LOC100130849
	LOC100131581
	LOC100132147
	LOC100132738
	LOC100144597
	LOC100289333

	LOC100291323<br>LOC100507634<br>LOC128322<br>LOC150568<br>LOC150935<br>LOC220980<br>LOC285370<br>LOC285778<br>LOC286190<br>LOC339862<br>LOC401433<br>LOC440117<br>LOC442028<br>LOC494558<br>LOC554201<br>LOC645591<br>LOC646034<br>LOC728084<br>LOC728192<br>LOC729177<br>LOC729444<br>LOC90834<br>LOC91149<br>LPHN3<br>LRRTM2<br>MAGEB5<br>MCHR2<br>MCTP2<br>MFSD6L<br>MGC12916<br>MNDA<br>MUC12<br>MYBL1<br>MYOCD<br>NCRNA00264<br>NCRNA00284<br>NCRNA00307<br>NDUFS7<br>NEB<br>NHLH1<br>NPIPL3<br>NT5DC4<br>NTNG1<br>ODF2L<br>OPN5<br>OR4C16<br>OR4D2<br>OR4F4<br>OR6C76<br>OR9G4<br>PABPC1P2<br>PIAS4<br>PLB1<br>PLCH1<br>PLP1<br>POM121L1P<br>POPDC2<br>PPP1R1C<br>PRAMEF13<br>PROZ<br>PRPS1<br>PSG9<br>RAD9B<br>RGR<br>RUNX2<br>SAMD12<br>SASH1

	SCNN1A<br>SEC14L3<br>SEMG2<br>SERPINI2<br>SFTPB<br>SHCBP1L<br>SLC14A1<br>SLC3A1<br>SNAR-C3<br>SNORA2B<br>SNORA77<br>SPANXB2<br>SPINT4<br>SST<br>ST8SIA6<br>STAP1<br>SULT1C2<br>SYCP2L<br>SYT6<br>TARP<br>TCEB3C<br>TCTN3<br>TDRD5<br>TFAP2D<br>THUMPD2<br>TIMP1<br>TPTE2<br>TRAF3IP3<br>TRAPPC10<br>TRIOBP<br>TRPM6<br>TTTY21<br>TTTY3<br>TTTY8<br>TUBBP5<br>TXNRD3NB<br>UBASH3A<br>UBE2D3<br>UBE4B<br>UGT1A4<br>UGT1A6<br>USH2A<br>WIPF3<br>ZBP1<br>ZCCHC11<br>ZNF167<br>ZNF354C<br>ZNF853<br>ZNF883<br>ZSCAN5B

Supplementary Table 2. Substrate compounds of CYP1A1 and CYP1B1 retrieved from cytochrome P450 database SuperCYP (http://bioinformatics.charite.de/supercyp/index.php?site=home, accessed through ‘CYP-drug interaction’ on 03.08.2020), and SLCO2B1 substrates retrieved from metabolism and transport database Metrabase (http://www-metrabase.ch.cam.ac.uk/, accessed through ‘search by protein’ on 03.08.2020).

Enzyme/ transporter	CYP1A1	CYP1B1	SLCO2B1
Substrates	Acriflavinium-chloride<br>Alum<br>Amiodarone<br>Amodiaquine<br>Benzydamine<br>Capsaicin<br>Chloroquine<br>Cinnarizine<br>Clenbuterol<br>Dacarbazine<br>Daunorubicin<br>Debrisoquine<br>Decamethrin<br>Diclofenac<br>Dronabinol<br>Erythromycin<br>Estradiol<br>Estrone<br>Etacrynic acid<br>Ethinylestradiol<br>Flunarizine<br>Flutamide<br>Fluvastatin<br>Granisetron<br>Haloperidol<br>Ipriflavone<br>Levothyroxine- sodium<br>Melatonin<br>Menadione<br>Mercaptopurine<br>Nicotine<br>Nitrofurantoin<br>Omeprazole<br>Oxaliplatin<br>Paracetamol<br>Perazine<br>Phenacetin<br>Prazosin<br>Procarbazine<br>Progesterone<br>Raltegravir<br>Retigabine<br>Riboflavin (vit B2)<br>Riluzole<br>Tamoxifen<br>Testosterone<br>Theophylline<br>Toremifene<br>Troglitazone<br>Zotepine	Amodiaquine<br>Caffeine<br>Dibutylphthalate<br>Docetaxel<br>Erythromycin<br>Estradiol<br>Estrone<br>Flutamide<br>Hydrogen-peroxide<br>Melatonin<br>Oxaliplatin<br>Procarbazine<br>Progesterone<br>Retinol (vit A)<br>Rosuvastatin<br>Testosterone<br>Theophylline	Dinoprostone<br>Penicillin G<br>Bromosulfophthalein disodium<br>Estrone-3-sulfate<br>Dehydroepiandrosterone sulfate<br>Taurocholic acid<br>Pravastatin<br>Glyburide<br>Pregnenolone sulfate<br>Aliskiren<br>Pemetrexed<br>Fluvastatin<br>Bosentan<br>Etoposide<br>Methanone, [4-[4-(3,4-dihydro-6,7-dimethoxy-2(1H)-isoquinolinyl)-6,7-dimethoxy-2-quinazolinyl]hexahydro-1H-1,4-diazepin-1-yl](3,4-dihydroxy-1-pyrrolidinyl)<br>Rocuronium<br>N-methyl-quinidine<br>Digoxin<br>Pitavastatin<br>Atorvastatin<br>Telmisartan<br>Rosuvastatin<br>CP 671305<br>M17055<br>Telmisartan glucuronide<br>Levothyroxine<br>Talinolol<br>Tebipenem pivoxil<br>BDE 47<br>BDE99<br>BDE153<br>Bromosulfophthalein<br>Latanoprost acid<br>Salozinal<br>3-Pyridinecarboxylic acid, 6-[[(2S)-3-cyclopentyl-1-oxo-2-[4-(trifluoromethyl)-1H-imidazol-1-yl]propyl]amino]-<br>Ethinylestradiol-3-O-sulfate<br>Cerivastatin<br>Montelukast<br>Sulfasalazine<br>Fexofenadine<br>Eltrombopag<br>(betaR,deltaR)-5-cyclopropyl-2-(4fluorophenyl)-4-[[[(3 fluorophenyl)methyl]amino]carbonyl]-beta,delta-dihydroxy-1H-imidazole-1-heptanoic acid<br>Unoprostone carboxylate<br>Ezetimibe glucuronide<br>Iloprost<br>Thromboxane B2

			Scutellarein 6-O-beta-D-glucuronide<br>Scutellarein 7-O-beta-D-glucuronide

Supplementary Table 3. Genes regulated (≥2 fold up or down) by 4 h Wnt5A/IL-1β combination treatment in HCAEC. Data are from 3 independent array experiments.

Upregulated genes	Downregulated genes
ABTB2	A2ML1
ADAM21	ACOT6
ADAMTS9	ACSM5
AGMO	ADRB1
AMOT	AGBL2
AMPD3	AJAP1
APOBEC3G	AKNA
APOL2	ALDOAP2
APOL3	ANK3
APOL4	ANKRD20A2
APOL6	ANKRD20A9P
ARHGAP9	ANKRD23
ARID5B	ANKRD5
ATF3	ANXA11
ATP2C2	AQP9
ATPBD4	ARHGAP18
BARHL1	ARHGEF4
BATF3	BCL11A
BCL2A1	BEST1
BCL6	BIN2
BDKRB2	C10orf99
BHLHE41	C12orf54
BICC1	C13orf15
BIRC3	C14orf23
BMP2	C18orf34
C10orf93	C1orf141
C11orf40	C1orf229
C11orf96	C5orf52
C12orf50	C5orf62
C12orf53	C7orf58
C14orf28	C9orf66
C15orf48	CACNA1G
C19orf29OS	CAGE1
C1orf172	CCDC108
C1QTNF1	CCDC121
C1R	CCDC165
C1S	CCDC48
C22orf33	CCNC
C2CD4A	CDC14B
C2CD4B	CDCA7L
C3	CEACAM7
C3orf52	CES1P1
C6	CH25H
C6orf58	CLEC4GP1
C7orf51	CMAHP
C8orf4	CNIH3
C8orf80	COL22A1
CACNA1B	COX7B2
CAMTA1	CSMD2
CBLN4	CXorf22
CBR3	CXorf51
CCL1	CYP26B1
CCL17	DCHS2
CCL2	DDIT4L
CCL20	DEFB113
CCL3	DLL4
CCL3L3	DNAJB4
CCL4	DNALI1
CCL5	DPP6
CCL7	DSPP
CCL8	E2F2

CCRN4L<br>CD69<br>CD7<br>CD70<br>CD83<br>CDHR3<br>CFB<br>CHDH<br>CHP2<br>CHRDL2<br>CHRNA2<br>CHST6<br>CITED4<br>CLDN1<br>CLDN14<br>CLEC2D<br>CLEC4A<br>CNOT4<br>CNTN2<br>COL27A1<br>CPEB4<br>CRIP1<br>CSF1<br>CSF2<br>CSF3<br>CSRP2<br>CTHRC1<br>CTSL1<br>CX3CL1<br>CXCL1<br>CXCL10<br>CXCL2<br>CXCL3<br>CXCL5<br>CXCL6<br>CXCR7<br>CYLD<br>CYP1A1<br>CYP1B1<br>CYP7A1<br>CYS1<br>DCUN1D2<br>DDX58<br>DENND2D<br>DIO3<br>DISC1<br>DLG2<br>DNAJB9<br>DNM1L<br>DRAM1<br>DSCR6<br>DUSP16<br>EBI3<br>EDNRA<br>EFNA1<br>EGFL6<br>EGOT<br>EGR2<br>EGR3<br>EGR4<br>ELOVL7<br>ENKUR<br>ERC2<br>ERO1LB<br>ETV3<br>ETV7<br>F3	E2F8<br>EBF1<br>EFCAB11<br>EHD3<br>EIF3E<br>ENAH<br>EPHA5<br>EPN3<br>EVI2B<br>FAM124B<br>FAM201A<br>FAM26D<br>FAM74A1<br>FGF12<br>FGFBP3<br>FGFR2<br>FILIP1<br>FLG2<br>FLJ25328<br>FLJ41484<br>FRY<br>GATA4<br>GFOD1<br>GIMAP7<br>GJA4<br>GKN2<br>GLIPR1L2<br>GNG2<br>GOLGA7B<br>GPC6<br>GPR116<br>H2AFB3<br>HOXB7<br>HRCT1<br>HS3ST1<br>HS6ST3<br>HTR1D<br>HYDIN<br>ICA1L<br>IGSF6<br>IL16<br>INHBB<br>ISL1<br>KCNC2<br>KHK<br>KIAA1239<br>KIF2B<br>LDB2<br>LHX9<br>LILRB4<br>LOC100128402<br>LOC100129413<br>LOC100129518<br>LOC100130849<br>LOC100131023<br>LOC100131395<br>LOC100131581<br>LOC100132147<br>LOC100507012<br>LOC100510059<br>LOC158376<br>LOC282980<br>LOC284080<br>LOC285954<br>LOC286068<br>LOC286071<br>LOC286367

FAM101A	LOC340094
FAM122C	LOC400927
FAM129A	LOC400958
FAM129C	LOC401847
FAM150B	LOC440117
FAM201B	LOC441179
FAM71F2	LOC729156
FCGR2A	LOC729444
FCRL2	LOC90834
FER1L6-AS1	LOC91149
FGF18	LRRTM2
FGL1	LYPD1
FILIP1L	MAP7
FLJ25363	MBP
FLJ31104	MCF2L
FOXF1	MCHR2
FOXJ1	MDFI
FSTL3	MEF2C
G0S2	MNDA
GBP1	MTUS1
GBP3	MUC12
GBP4	MYCN
GBP5	NCRNA00284
GCH1	NCRNA00320
GFPT2	NEB
GK	NFIB
GOT1L1	NPIPL3
GPR37L1	NTNG1
GPR68	ODF2L
GRAMD3	OR2K2
GRK1	OR2T12
GRM4	OR5L1
HAS3	OR9G4
HBS1L	OTX1
HCG4B	OVOS2
HERC6	PALMD
HEY1	PAQR4
HGF	PARP15
HIVEP2	PCDH11X
HLA-B	PCDH15
HLA-C	PLAC8
HLA-F	PLB1
HOMER1	PLEKHG4B
ICAM1	PPFIBP2
ICAM4	PRB4
ICAM5	PRICKLE1
ICOSLG	PRO0611
IDO1	PRO1596
IER3	PRO1768
IFI30	PRODH
IFIH1	PRPS1
IFIT5	RASGRP3
IFNGR1	RASSF5
IFNGR2	RASSF9
IL10RA	RD3
IL15	RESP18
IL15RA	RFX4
IL18R1	RIN1
IL18RAP	SAMD13
IL1A	SEC14L3
IL1B	SEMG2
IL22RA2	SERTAD4
IL27	SFTPB
IL36G	SGCD
IL6	SGK223
IL8	SH3BP4
INHBA	SH3TC2

IQCA1<br>IRAK2<br>IRF1<br>IRX2<br>ISG20<br>ITGA1<br>ITGA4<br>JAM2<br>KCNE4<br>KCNN2<br>KDM6B<br>KIAA0247<br>KIAA1644<br>KIRREL2<br>KLF6<br>KLK3<br>KRT24<br>KRTAP3-2<br>KYNU<br>LAD1<br>LAMB3<br>LAMC2<br>LIF<br>LITAF<br>LOC100128126<br>LOC100128262<br>LOC100128371<br>LOC100128869<br>LOC100129104<br>LOC100131733<br>LOC100507410<br>LOC148145<br>LOC283352<br>LOC283922<br>LOC284263<br>LOC284570<br>LOC285972<br>LOC393076<br>LOC440896<br>LOC642620<br>LOC646999<br>LOC728228<br>LOC728752<br>LTB<br>LYPD6<br>LYSMD3<br>MAGEC2<br>MAN1B1<br>MAP3K8<br>MCM9<br>MDGA1<br>MIR155HG<br>MMP1<br>MMP10<br>MRGPRX3<br>MS4A2<br>MSX1<br>MTHFD2L<br>MTMR7<br>MUC4<br>MYB<br>MYOM1<br>N4BP2L1<br>NAB1<br>NAMPT<br>NCOA7<br>NEURL3	SHE<br>SIRPD<br>SKOR1<br>SLC22A2<br>SLC4A10<br>SMAD6<br>SNAP91<br>SNTG1<br>SOX18<br>SPANXN3<br>SPIC<br>SRRM3<br>ST8SIA4<br>ST8SIA6<br>SULT1C2<br>SYNPO2<br>TBX1<br>TEX15<br>THBD<br>TMC1<br>TMEM100<br>TPD52L1<br>TRIM36<br>TTN<br>TTTY21<br>TUBB1<br>TUBBP5<br>WDFY4<br>WWTR1<br>XKR4<br>YIF1B<br>ZCCHC11<br>ZDHHC15<br>ZNF30<br>ZNF365<br>ZNF367<br>ZNF395

NFKB1<br>NFKBIA<br>NFKBID<br>NFKBIZ<br>NINJ1<br>NIPAL4<br>NKAIN1<br>NKD2<br>NKX3-1<br>NNMT<br>NOD2<br>NPTX1<br>NR4A3<br>NR6A1<br>NRIP1<br>NTN1<br>NUAK2<br>NUP62CL<br>NUPL1<br>OR10H1<br>OR11H12<br>OR1F1<br>OR2M2<br>OR5M11<br>OR5P2<br>OSGIN2<br>OXTR<br>P2RY6<br>PARD3<br>PARP14<br>PDE4DIP<br>PDE5A<br>PDGFRA<br>PDLIM4<br>PDPR<br>PDZD2<br>PEG10<br>PELI1<br>PGR<br>PITPNC1<br>PITX2<br>PLA1A<br>PLA2G4A<br>PLCB4<br>POU5F2<br>PPAP2B<br>PRINS<br>PRRX1<br>PSMD5<br>PTGFR<br>PTGS2<br>PTPRC<br>PTX3<br>RAB11FIP4<br>RASGRP1<br>RASSF3<br>RC3H1<br>RCAN1<br>RCSD1<br>REL<br>RELB<br>RFPL4B<br>RGS16<br>RGS2<br>RIPK2<br>RLBP1<br>RND1	

RNF157<br>RRAD<br>RSPO3<br>RTP4<br>RUNX1<br>S100A3<br>SAMD4A<br>SAMD9L<br>SAT1<br>SAV1<br>SDC4<br>SEC24A<br>SELE<br>SEMA3C<br>SERPINA3<br>SERPINB13<br>SERPINB2<br>SERPINE2<br>SGPP2<br>SHANK2<br>SLC10A7<br>SLC12A7<br>SLC19A2<br>SLC22A24<br>SLC2A6<br>SLC30A3<br>SLC41A2<br>SLC44A3<br>SLC6A4<br>SLC7A2<br>SLC8A3<br>SLC9A4<br>SLCO4A1<br>SLCO5A1<br>SNORA60<br>SNX3<br>SOCS1<br>SOD2<br>SOX3<br>SPINLW1<br>SPON2<br>SQRDL<br>SRD5A2<br>SSU72<br>ST6GAL1<br>ST8SIA2<br>STAT5A<br>STC2<br>STON2<br>SYT9<br>TAP1<br>TAS2R39<br>TCHH<br>TFAP2A<br>TGFB3<br>THSD7B<br>TIAM2<br>TIFA<br>TLR2<br>TM4SF4<br>TMCO2<br>TMEM106A<br>TMEM146<br>TMEM217<br>TMEM37<br>TMEM71<br>TMEM72	

TNF<br>TNFAIP2<br>TNFAIP3<br>TNFAIP6<br>TNFAIP8<br>TNFRSF11B<br>TNFRSF9<br>TNFSF10<br>TNFSF15<br>TNFSF9<br>TNIP1<br>TNIP3<br>TPPP<br>TRAF1<br>TRIM36<br>TSLP<br>TTC30A<br>TTC39A<br>UBD<br>UBR4<br>UGCG<br>UMODL1<br>UPP1<br>VCAM1<br>VEGFA<br>VIPR2<br>VSTM1<br>WNT7B<br>WTAP<br>ZC3H12A<br>ZC3H12C<br>ZDHHC23<br>ZFR2<br>ZNF785	

Supplementary Table 4. Regulated genes of drug and xenobiotic metabolism pathways significantly *(P<0.05)* enriched in 4 h Wnt5A+IL-1β transcriptome of HCAEC. Data are from 3 independent array experiments.

Gene symbol	Protein name	Class	Regulation
†ABTB2[a]	Ankyrin repeat and BTB/POZ domain containing protein 2	Generic binding protein	Up
†AMPD3[a]	AMP deaminase 3	Generic enzyme	Up
†AQP9[e]	Aquaporin-9	Generic channel	Down
†BCL2A1[a]	Bcl-2-related protein A1	Generic binding protein	Up
†BDKRB2[a,b,c]	B2 bradykinin receptor	GPCR	Up
†C1R[b]	Complement C1r subcomponent	Generic protease	Up
†C1S[b]	Complement C1s subcomponent	Generic protease	Up
†C3[b]	Complement C3	Generic binding protein	Up
†CACNA1B[a]	Voltage-dependent N-type calcium channel subunit alpha-1B	Voltage-gated ion-channel	Up
CCL1[a]	C-C motif chemokine 1	Receptor ligand	Up
†CCL2[a,c]	C-C motif chemokine 2	Receptor ligand	Up
†CCL20[a]	C-C motif chemokine 20	Receptor ligand	Up
†CCL4[a]	C-C motif chemokine 4	Receptor ligand	Up
†CCL5[a]	C-C motif chemokine 5	Receptor ligand	Up
CCL7[a]	C-C motif chemokine 7	Receptor ligand	Up
†CCL8[b]	C-C motif chemokine 8	Receptor ligand	Up
†CFB[b]	Complement factor B	Generic protease	Up
COX7B2[a,b]	Cytochrome c oxidase subunit 7B2, mitochondrial	Generic enzyme	Down
CSF2[b]	Granulocyte-macrophage colony-stimulating factor	Receptor ligand	Up
†CSF3[b]	Granulocyte-macrophage colony-stimulating factor	Receptor ligand	Up
†CTSL[b]	Cathepsin L1	Generic protease	Up
†CXCL10[b]	C-X-C motif chemokine 10	Receptor ligand	Up
*†CYP1A1[a,c,f,g]	Cytochrome P450 1A1	Generic enzyme	Up
≠†CYP7A1[b,c,d,f]	Cytochrome P450 7A1	Generic enzyme	Up
†CYP1B1[a,b,g]	Cytochrome P450 1B1	Generic enzyme	Up
DCHS2[a]	Protocadherin-23	Generic binding protein	Down
†DNALI1[b]	Axonemal dynein light intermediate polypeptide 1	Generic binding protein	Down
EBI3[a]	Interleukin-27 subunit beta	Receptor ligand	Up
*†EDNRA[a,b,c]	Endothelin-1 receptor	GPCR	Up
†EFNA1[b]	Ephrin-A1	Receptor ligand	Up
≠†EPHA5[c]	Ephrin type-A receptor 5	Receptor with enzyme activity	Down
†FCGR2A[a]	Low affinity immunoglobulin gamma Fc region receptor II-a	Generic receptor	Up
†FILIP1L[b]	Filamin A-interacting protein 1-like	Generic protein	Up
†GBP1[a,b]	Guanylate-binding protein 1	Generic binding protein	Up
†GCH1[a]	GTP cyclohydrolase 1	Generic enzyme	Up
≠†GNG2[a,b]	Guanine nucleotide-binding protein G(I)/G(S)/G(O) subunit gamma-2	G beta/gamma	Down
†GPR37L1[a,b,c]	G-protein coupled receptor 37-like 1	GPCR	Up
≠†HOXB7[a]	Homeobox protein Hox-B7	Transcription factor	Down
†ICAM1[a,c,d]	Intercellular adhesion molecule 1	Generic receptor	Up
†IDO1[a,b]	Indoleamine 2,3-dioxygenase 1	Generic enzyme	Up
≠†IFNGR1[a]	Interferon gamma receptor 1	Generic receptor	Up
≠†IFNGR2[a]	Interferon gamma receptor 2	Generic receptor	Up
†IL1B[a,b,c,d]	Interleukin-1 beta	Receptor ligand	Up
†IL6[a,b,c,d]	Interleukin-6	Receptor ligand	Up
IL10RA[b]	Interleukin-10 receptor subunit alpha	Generic receptor	Up
†IRF1[a]	Interferon regulatory factor 1	Transcription factor	Up
†ITGA4[a]	Integrin alpha-4	Generic receptor	Up
†KDM6B[b]	Lysine-specific demethylase 6B	Generic enzyme	Up
†KLF6[a]	Krueppel-like factor 6	Transcription factor	Up
≠KLK3[b,f]	Prostate-specific antigen	Generic protease	Up
†LAMB3[c]	Laminin subunit beta-3	Receptor ligand	Up
†LAMC2[c]	Laminin subunit gamma-2	Receptor ligand	Up

†LILRB4[b]	Leukocyte immunoglobulin-like receptor subfamily B member 4	Generic receptor	Down
†MS4A2[b]	High affinity immunoglobulin epsilon receptor subunit beta	Generic receptor	Up
†MTMR7 [a]	Myotubularin-related protein 7	Protein phosphatase	Up
MYB[c]	Transcriptional activator Myb	Transcription factor	Up
†NAB1 [a]	NGFI-A-binding protein 1	Transcription factor	Up
†NCOA7 [a]	Nuclear receptor coactivator 7	Generic binding protein	Up
†NFIB [a]	Nuclear factor 1 B-type	Transcription factor	Down
†NFKB1 [a]	Nuclear factor NF-kappa-B p105 subunit	Transcription factor	Up
†NINJ1 [a,b]	Ninjurin-1	Generic binding protein	Up
†NNMT[b,h]	Nicotinamide N-methyltransferase	Generic enzyme	Up
†NR4A3[b]	Nuclear receptor subfamily 4 group A member 3	Transcription factor	Up
†NTN1 [a]	Netrin-1	Receptor ligand	Up
OR10H1[c]	Olfactory receptor 10H1	GPCR	Up
OR1F1[c]	Olfactory receptor 1F1	GPCR	Up
OR2K2[c]	Olfactory receptor 2K2	GPCR	Down
OR2M2[c]	Olfactory receptor 2M2	GPCR	Up
OR5L1[c]	Olfactory receptor 5L1	GPCR	Down
OR5M11[c]	Olfactory receptor 5M11	GPCR	Up
OR5P2[c]	Olfactory receptor 5P2	GPCR	Up
*OR9G4[c]	Olfactory receptor 9G4	GPCR	Down
OXTR [a,b,c]	Oxytocin receptor	GPCR	Up
†PDE5A [a]	cGMP-specific 3',5'-cyclic phosphodiesterase	Generic enzyme	Up
†PDGFRA [a,b,d]	Platelet-derived growth factor receptor alpha	Receptor with enzyme activity	Up
†PDLIM4[b]	PDZ and LIM domain protein 4	Generic binding protein	Up
†PEG10 [a]	Retrotransposon-derived protein PEG10	Generic binding protein	Up
†PGR [a]	Progesterone receptor	Transcription factor	Up
≠†PLA2G4A [a,b,g]	Cytosolic phospholipase A2	Generic phospholipase	Up
†PTGS2 [a,b,d]	Prostaglandin G/H synthase 2	Generic enzyme	Up
†REL [a]	Proto-oncogene c-Rel	Transcription factor	Up
†RELB [a]	Transcription factor RelB	Transcription factor	Up
≠RESP18 [a]	Regulated endocrine-specific protein 18	Generic protein	Down
†SAT1[b]	Diamine acetyltransferase 1	Generic enzyme	Up
†SDC4 [a,b]	Syndecan-4	Generic receptor	Up
SLC22A2[e]	Solute carrier family 22 member 2	Transporter	Down
SLC22A24[e]	Solute carrier family 22 member 24	Transporter	Up
SLC6A4[e]	Sodium-dependent serotonin transporter	Transporter	Up
†SLC7A2[e]	Cationic amino acid transporter 2	Transporter	Up
†SLCO4A1[e]	Solute carrier organic anion transporter family member 4A1	Transporter	Up
†SOCS1 [a]	Suppressor of cytokine signaling 1	Generic binding protein	Up
†SOD2 [a]	Superoxide dismutase [Mn], mitochondrial	Generic enzyme	Up
†STAT5A [a]	Signal transducer and activator of transcription 5A	Transcription factor	Up
†STC2 [a]	Stanniocalcin-2	Generic binding protein	Up
†SYT9 [a]	Synaptotagmin-9	Generic receptor	Up
TFAP2A[d]	Transcription factor AP-2-alpha	Transcription factor	Up
≠†TGFB3 [a,b]	Transforming growth factor beta-3 proprotein	Receptor ligand	Up
†TLR2 [a]	Toll-like receptor 2	Generic receptor	Up
†TNF [a,b,c,d]	Tumor necrosis factor	Receptor ligand	Up
†TNFAIP2[b]	Tumor necrosis factor alpha-induced protein 2	Generic protein	Up
≠†TNFRSF9[b]	Tumor necrosis factor receptor superfamily member 9	Generic receptor	Up
†TNFSF10[f]	Tumor necrosis factor ligand superfamily member 10	Receptor ligand	Up
†VCAM1 [a]	Vascular cell adhesion protein 1	Generic receptor	Up
≠†VEGFA[b]	Vascular endothelial growth factor A	Receptor ligand	Up
WNT7B [a]	Protein Wnt-7b	Receptor ligand	Up

	WW domain-containing transcription regulator protein 1		

Genes regulated in AhR mediated regulation_heart[a]
Genes regulated in LXR mediated regulation_heart[b]
Genes regulated in PXR mediated regulation_heart[c]
Genes regulated in FXR mediated regulation_heart[d]
Genes regulated in Xenobiotic Metabolism. Phase III_heart[e]
Genes regulated in CAR mediated regulation_heart[f]
Genes regulated in Xenobiotic Metabolism. Phase I_heart[g]
Genes regulated in Xenobiotic Metabolism. Phase II_heart[h]
*similarly regulated across Wnt5A, Wnt5A+IL-1, and IL-1[$] transcriptomes
≠regulated only in Wnt5A+IL-1 but not in Wnt5A or IL-1[$] transcriptomes
[†]Protein expression verified in normal human myocardium as shown in The Human Protein Atlas database (accessed on 03.08.2020)
[$]Genes of Drug and xenobiotic metabolism pathways most significantly (*P<0.05*) regulated by 4 h IL-1β treatment in HCAEC (Refer Supplementary Figure 1). Genes were identified by reanalysing data published in *Skaria, T., Bachli, E., and Schoedon, G. (2019). Gene Ontology Analysis for Drug Targets of the Whole Genome Transcriptome of Human Vascular Endothelial Cells in Response to Proinflammatory IL-1. Frontiers in Pharmacology 10*. The purpose of reanalysing data from above-said study was to identify the genes of myocardial drug and xenobiotic metabolism pathways regulated by 4 h IL-1β treatment (which was not contained in previous study) that served as an additional positive control for comparing the effects of Wnt5A/ IL-1β combination treatment with sole Wnt5A treatment in HCAEC.

Supplementary Figure 1

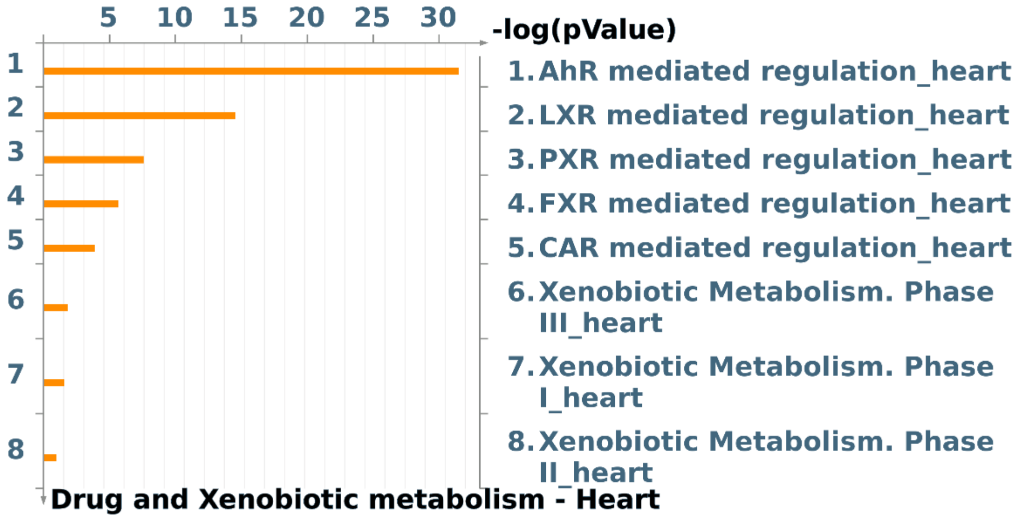

Supplementary Figure 1. Drug and xenobiotic metabolism pathways most significantly *(P<0.05)* regulated by 4 h IL-1β treatment in HCAEC. Pathways represented as histograms are ranked by the –log value (P value). This figure is generated by reanalysing data published in *Skaria, T., Bachli, E., and Schoedon, G. (2019). Gene Ontology Analysis for Drug Targets of the Whole Genome Transcriptome of Human Vascular Endothelial Cells in Response to Proinflammatory IL-1. Frontiers in Pharmacology 10*. The purpose of reanalysing data from above-said study was to identify the myocardial drug and xenobiotic metabolism pathways regulated by 4 h IL-1β treatment (which was not contained in previous study) that served as an additional positive control for comparing the effects of Wnt5A/ IL-1β combination treatment with sole Wnt5A treatment in HCAEC.
